# Participant characteristics and reasons for non-consent to health information linkage for research: experiences from the ATHENA COVID-19 study

**DOI:** 10.1186/s12911-023-02370-6

**Published:** 2024-01-23

**Authors:** Kim Greaves, Amanda King, Zoltan Bourne, Jennifer Welsh, Mark Morgan, M. Ximena Tolosa, Carissa Bonner, Tony Stanton, Michael Fryer, Rosemary Korda

**Affiliations:** 1 Sunshine Coast University Hospital, Queensland Health, 6 Doherty Street, Birtinya, QLD 4575 Australia; 2 National Centre for Epidemiology and Population Health, ANU College of Health and Medicine, Australian National University, Canberra, ACT 2600 Australia; 3 Hinterland Health, Maple Street, Maleny, QLD 4551 Australia; 4 Faculty of Health Sciences and Medicine, Bond University, Gold Coast, QLD 4229 Australia; 5 Queensland Department of Health, Communicable Disease Branch, 15 Butterfield St, Herston, QLD 4001 Australia; 6 School of Public Health, Sydney Medical School, University of Sydney, Sydney, NSW 2006 Australia

**Keywords:** Primary health care, Data linkage, Sharing, Non-consent, Recontact, ATHENA, COVID-19

## Abstract

**Background:**

The linkage of primary care, hospital and other health registry data is a global goal, and a consent-based approach is often used. Understanding the attitudes of why participants take part is important, yet little is known about reasons for non-participation. The ATHENA COVID-19 feasibility study investigated: 1) health outcomes of people diagnosed with COVID-19 in Queensland, Australia through primary care health data linkage using consent, and 2) created a cohort of patients willing to be re-contacted in future to participate in clinical trials. This report describes the characteristics of participants declining to participate and reasons for non-consent.

**Methods:**

Patients diagnosed with COVID-19 from January 1^st^, 2020, to December 31^st^, 2020, were invited to consent to having their primary healthcare data extracted from their GP into a Queensland Health database and linked to other data sets for ethically approved research. Patients were also asked to consent to future recontact for participation in clinical trials. Outcome measures were proportions of patients consenting to data extraction, permission to recontact, and reason for consent decline.

**Results:**

Nine hundred and ninety-five participants were approached and 842(85%) reached a consent decision. 581(69%), 615(73%) and 629(75%) consented to data extraction, recontact, or both, respectively. Mean (range) age of consenters and non-consenters were 50.6(22-77) and 46.1(22-77) years, respectively. Adjusting for age, gender and remoteness, older participants were more likely to consent than younger (aOR 1.02, 95%CI 1.01 to 1.03). The least socio-economically disadvantaged were more likely to consent than the most disadvantaged (aOR 2.20, 95% 1.33 to 3.64). There was no difference in consent proportions regarding gender or living in more remote regions. The main reasons for non-consent were ‘not interested in research’ (37%), ‘concerns about privacy’ (15%), ‘not registered with a GP’ (8%) and ‘too busy/no time’ (7%). ‘No reason’ was given in 20%.

**Conclusion:**

Younger participants and the more socio-economically deprived are more likely to non-consent to primary care data linkage. Lack of patient interest in research, time required to participate and privacy concerns, were the most common reasons cited for non-consent. Future health care data linkage studies addressing these issues may prove helpful.

## Background

The improvement of healthcare delivery through the linkage of primary care, hospital and other health registry data is a global goal [1]. These linked data can be used for research, including the development of cohort studies and, provided consent for recontact is obtained, can be an efficient means of recruitment of participants to clinical trials investigating new diagnostic tests or treatments [2–5]. Primary care data is particularly valuable as it contains detailed information on an individual’s risk factors, health measurements and treatments. Furthermore, this information is available at scale, with an estimated 80% of Australians visiting a general practitioner at least once in a year [6]. However, Australia has lagged behind other high-income nations in linking primary care data to other regional and national sources [7]. Reasons for this include general practice (GP) clinics working as private companies and use of heterogenous software platforms that are not interoperable with each other or other data repositories [8–10].

Large epidemiological surveys frequently seek consent to link health data with other datasets [11]. Although some studies employ opt-out consent for these linkages, an opt-in consent for linkage has also been used as this greatly reduces the onus on researchers to demonstrate the benefit to public interest if they have used opt-out consent [12]. A 2022 narrative review comprising 27 qualitative and quantitative studies, as well as systematic reviews, examined patients' and the general public's perspectives on the sharing of health data for research, including that of data linkage [13]. The findings revealed a significant level of public support for data sharing, albeit with certain conditions. Primarily, individuals emphasised the importance of providing information and ensuring transparency about data sharing procedures. Additionally, they expressed a desire to maintain some level of control over the sharing of their data. As a result, the review suggested the potential necessity of a consent process being required to meet these conditions. This was despite participants recognising that seeking consent could introduce bias due to potentially lower consent proportions. However, seeking consent does not always result in low consent proportions. A systematic review of the proportions of patients consenting to data linkage found the consent proportion varied widely from 39% up to 97% [14]. Therefore, to link and use primary care data for research it is essential that as large a number of participants participate as possible. If large numbers of people do not consent, it may increase bias and undermine statistical power. For example, in one study, those who gave consent (vs non-consent) were more likely to be older, in poorer health and to use health services more [15]. Another study linking survey data with administrative data found that older age and being employed had higher consent proportions to linkage [16]. It is therefore imperative that we understand the attitudes of participants whom we are approaching for consent. Whilst numerous studies have examined the motives and experiences of individuals who participate in research, little is known about reasons for non-participation, particularly in an Australian context [17]. Understanding these reasons is important if we are to attempt to improve consent rates for data linkage for the purposes of research in Australia.

The ATHENA (Australians Together Health Initiative) COVID-19 (Coronavirus disease 2019) (ACV19) study was set up to enable ongoing investigation of health outcomes for all people diagnosed with COVID-19 in Queensland, Australia. Using informed consent, primary healthcare data was linked with other administrative data sources for the purposes of research [18]. The study was also designed to recruit a cohort of participants who had had COVID-19 with linked health data willing to be contacted in future to participate in clinical trials. The experiences gained from this study would also inform the future implementation of the ATHENA program, a state-wide initiative that uses dynamic consent for mass recruitment of participants for clinical trials and other research [19]. This study reports on the proportion of participants agreeing to health care data linkage for research purposes and permission for re-contact, the characteristics of participants and their reasons for non-consent when asked to take part in the ATHENA COVID-19 study.

## Methods

The Queensland Health funded ATHENA program involves the integration of primary health care, hospital, and other healthcare data sets across Queensland. The vision of the ATHENA program is to establish a state-wide registry containing the healthcare information and biospecimens of several million Australians across Queensland using dynamic consent to connect participants, researchers, and the clinical trial industry. The goal is to accelerate growth in cutting-edge commercial and non-profit health research. This will result in health and economic benefits for both Queensland and other states. As part of this, the ACV19 study was set up in early 2020 in response to the COVID-19 pandemic to create a cohort of people diagnosed with COVID-19 in Queensland with linked primary (general practice) health care, hospital, and registry data. There were two parts to the study. Part 1 (completed, described in detail elsewhere) linked Queensland COVID-19 hospital and administrative data including notifiable conditions and death registrations, and did not require informed consent [18]. Access to patient contact details and other health information for Part 1 was granted under Section 282 of the Public Health Act *2005*. Part 2 (described here) linked data from Part 1 to participants’ healthcare information held by GPs and required patient consent*.* All participants notified to the Queensland Department of Health as having a COVID-19 (defined as testing positive for the Severe Acute Respiratory Syndrome Coronavirus 2 virus in Queensland) in the period January 1^st^ 2020 to December 31^st^ 2020 were contacted to gain consent to have their primary healthcare data extracted from their GP into a Queensland Health database and linked to other data sets for ethically approved COVID-19 research. Participants were also asked for consent to be recontacted in future to discuss participation in clinical trials.

### Patient recruitment and recording of responses

Participants were identified as having had COVID-19 from the Queensland Health’s Notifiable Conditions System (NOCS). Patients who lived overseas or interstate were excluded as it was not possible to link their data. Participants were telephoned by an ACV19 liaison team member to ask permission to email or post them an information pack about the study. The liaison team consisted of five staff: a general practitioner (team lead), two registered nurses, a Primary Health Network (PHN) practice support officer and an allied health worker. Once sent, after a period of 2–5 days the patient was recontacted to answer any further questions and guide them through the Patient Information Consent Form (PICF). Participants were asked to electronically consent or decline the PICF using DocuSign or, manually sign-and-return the surface-mailed PICF in the reply-paid envelope. Participants were requested to identify a maximum of three regular GPs they had either currently or previously consulted. It is worth noting that in Australia, individuals have the option to consult multiple practices. Participants were also informed that their consent was being verbally recorded. If patients declined to consent, they were asked to volunteer a reason, which was recorded. Patients who did not respond to the second contact were sent a text reminder which they were able to respond by text and if they wished, give reasons why they declined. Patients who could not be contacted initially or recontacted, were excluded from the final study cohort. Prior to patient recruitment, the ACV19 study was advertised in PHN newsletters. A page on the Queensland Health website was created for the ACV19 study, which was publicly accessible to GPs and participants [20]. The site contained study information and supporting letters from the RACGP and all seven PHNs in Queensland.

### General practice interaction

For those participants who consented for data extraction, their GPs were contacted by the liaison team and a request made for export of the identifiable whole patient file to the Queensland Health ACV19 database. Primary healthcare data were linked probabilistically, using name, date of birth and address by the Statistical Services Branch within Queensland Health, to the Queensland COVID-19 hospital and administrative data including COVID-19 notifications and death registrations from Part 1, using established protocols [21].

### Data

To manage tracking and flow of patient recruitment and GP interactions, an electronic clinical trials management system was used (Clinical Conductor). The liaison team recorded all patient and GP responses and interactions in the clinical trial management system. Once the study was completed the responses were reviewed over multiple inductive cycles and broad general themes were developed. From these and known patient attitudes to sharing data published in the literature, a list of categories was created for the reasons given for declining consent. These were: 1) not interested in research; 2) no reason given/not disclosed; 3) privacy concerns; 4) not having a regular GP 5) too busy/no time 6) other [22].

The liaison team were encouraged wherever possible to record patient quotes verbatim to substantiate the category chosen. To identify patterns from interactions with the participants, a thematic analysis was conducted using a standard phased approach as a guide [23].

### Sociodemographic characteristics

Participant characteristics were derived from the NOCS register and included age, sex, remoteness (measured with Accessibility and Remoteness Index of Australia [ARIA +]), and socioeconomic status (measured using Socio-Economic Indexes for Areas, Index of Relative Socio-Economic Disadvantage, [SEIFA IRSD]) [24, 25]. ARIA + is a geographical measure of service accessibility based on road distances to service centres (based on population size), which groups areas into major cities; inner regional; outer regional; remote; and very remote areas. SEIFA IRSD is an area-based measure of socioeconomic status, based on average characteristics of the people living within areas containing around 10,000 people. A lower score indicates relatively greater disadvantage in general than a higher score. For this study, SEIFA IRSD scores were grouped into quartiles.

### Analysis

A descriptive analysis of factors potentially associated with non-consent was undertaken. The construction of the regression model was dictated by factors known to be associated with non-consent in the published literature and the variables available within the Notifiable Conditions System database [17, 26–29]*.* Univariate analysis was performed for each variable in relation to the outcome using odds ratios. A liberal p-value of 0.25 was considered as the cut-off for entry into the multivariate model. After the initial analysis gender didn’t meet these criteria, however as this was known to be a scientifically relevant variable it remained in the model.

We used the Hosmer–Lemeshow test, plots from residuals vs fitted values, standardised Pearson residuals and the Delta-Beta influence statistic to examine model fit. No interactions were noted between variables. Gender was not observed to be contributing significantly to the multivariate model, however we elected to retain the variable. The assumption of linearity in the logit for Age was confirmed.

We used logistic regression to quantify associations between “consent to data extraction for research” (binary outcome, Yes/No) and the sociodemographic characteristics. Age was treated as a continuous variable, while SEIFA IRSD, ARIA + and gender were treated as categorical variables.. Less than 2% of the data entries for ARIA + were missing. No other missing data entries were noted for the other variables. Given the low percentage of missing values they were assigned their own category that was included in the overall models. Effect sizes were reported as odds ratios with 95% confidence intervals.

All calculations were performed in Stata, version 16.1 (StataCorp, College Station, Tx, USA).

### Ethics approval

The ACV19 study was granted ethics approval by the Gold Coast Human Research Ethics Committee (HREC/2020/QGC/63555).

## Results

**Fig. 1:**
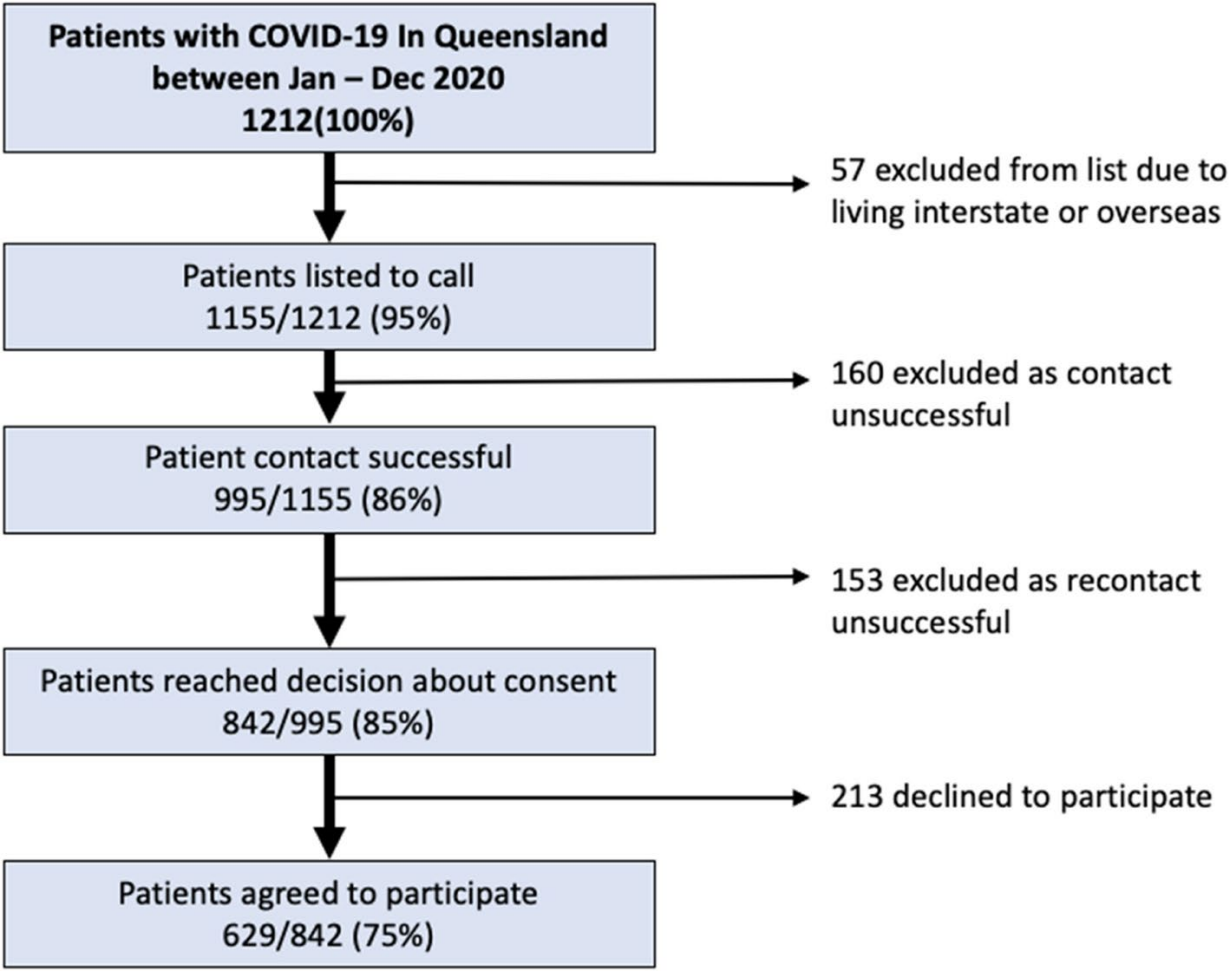
Flow chart of patient recruitment to the ATHENA COVID-19 study

A total of 1212 participants were registered in the Notifiable Conditions System as being reported as a COVID-19 case, from January 1^st^ 2020 to Dec 31^st^ 2020 in Queensland (Fig. 1). From these, 57 patients were removed from the list as they were living interstate or overseas, leaving 1157 patients listed to call. 995(86%) of these were successfully contacted and given information about the study, of whom 842(85%) responded with a consent decision and formed the final overall cohort for the study. 629(75%) consented to either health data linkage and/or to recontact to discuss clinical trial participation. Table 1 shows that of these, 581(69%) consented to health information linkage for research, and 261(31%) declined. Six-hundred and fifteen participants (73%) agreed to future recontact and 227(27%) declined. The mean age of the overall cohort was 49.2 (95% range 21—77) years, and 50% were male. The mean age of those who consented was 50.6 (95% range 22—77) years and declined 46.1 (95% range 20 -77) years.

**Table 1:** Categories of patient consent responses to health information linkage and use for research, and recontact to discuss participation in future clinical trials

	**Consent to health information linkage for research**			
**Consent to recontact**		**Agreed**	**Declined**	**Total**
	**Agreed**	567(67%)	48(6%)	615(73%)
	**Declined**	14(2%)	213(25%)	227(27%)
	**Total**	581(69%)	261(31%)	842

Of those who reached a consent decision, 73% lived in a major city and 26% were categorised as being in the most socioeconomically disadvantaged quartile. Table 2 shows that in all models’, increasing age was associated with a greater likelihood to consent to linkage. For example, adjusting for age, gender, and remoteness per 10-year increase in age, the odds of a person consenting were 1.2 times that of someone 10 years younger (aOR 1.02, 95%CI 1.01 to 1.03). In all models, those in the least socio-economically disadvantaged quartiles were more likely to consent than the most disadvantaged. For example, when adjusting for age, gender and remoteness, the odds of a person in the least disadvantaged quintile consenting was 2.2 times that of someone in the most disadvantaged quintile (aOR 2.20, 95% 1.33 to 3.64). There was no difference in consent proportions regarding gender or those living in more remote regions. The Hosmer–Lemeshow test showed high concordance between observed and expected responses, *p* = 0.97. Plots from residuals vs fitted values, standardised Pearson residuals and the Delta-Beta influence statistic were satisfactory**.**

**Table 2:** Proportions and odds ratios of patients who consented to primary health care data extraction and linkage for research, in relation to key socio-demographic characteristics

	Consent to data extraction for research	Model 1 OR (95% CI)	Model 2 OR (95% CI)	Model 3 OR (95% CI)
	Agreed N (%)			
Age (years)				
% increase per year^*^	581/842 (69%)	1.01 (1.00 – 1.02)^a^	1.02 (1.01 – 1.03)^c^	1.02 (1.01 – 1.03)^c^
Gender				
Male	288/421 (68.4)	1.00	1.00	1.00
Female	293/421 (69.6)	1.12 (0.83 – 1.50)	1.09 (0.80 – 1.47)	1.08 (0.80 – 1.46)
ARIA				
Major cities	427/616 (69.3)	1.00	-	1.00
Inner regional	105/149 (70.5)	0.76 (0.49 – 1.16)	-	0.86 (0.55 – 1.35)
Outer regional / remote / very remote	42/63 (66.7)	0.79 (0.44 – 1.39)	-	0.98 (0.54 – 1.80)
Missing	7/14 (50)	-	-	-
SEIFA IRSD				
Most Disadvantaged	137/215 (63.7)	1.00	1.00	-
2^nd^ quartile	164/221 (72.6)	1.84 (1.20 – 2.83)^b^	1.88 (1.21 – 2.91)^b^	-
3^rd^ quartile	127/197 (65.9)	1.38 (0.89 – 2.13)	1.41 (0.88 – 2.25)	-
Least Disadvantaged	153/209 (72.7)	1.97 (1.24 – 3.14)^d^	2.20 (1.33 – 3.64)^e^	-

Model 1 is adjusted for age and sex. Model 2 is adjusted for age, sex, and remoteness, measured with ARIA + . Model 3 is adjusted for age, sex, remoteness, and socioeconomic index. Age, sex, remoteness, and SEIFA IRSD are measured with or derived from NOCS data. SEIFA IRSD is measured in population-based quartiles. ^a^ *p* = 0.001; ^b^ *p* = 0.005; ^c^ *p* < 0.001; ^d^ *p* = 0.004; ^e^ *p* = 0.002

^*^For every 1 year increase in age, the odds of consenting to data extraction increased by 1% in model 1, and 2% in models 2 and 3

**Fig. 2:**
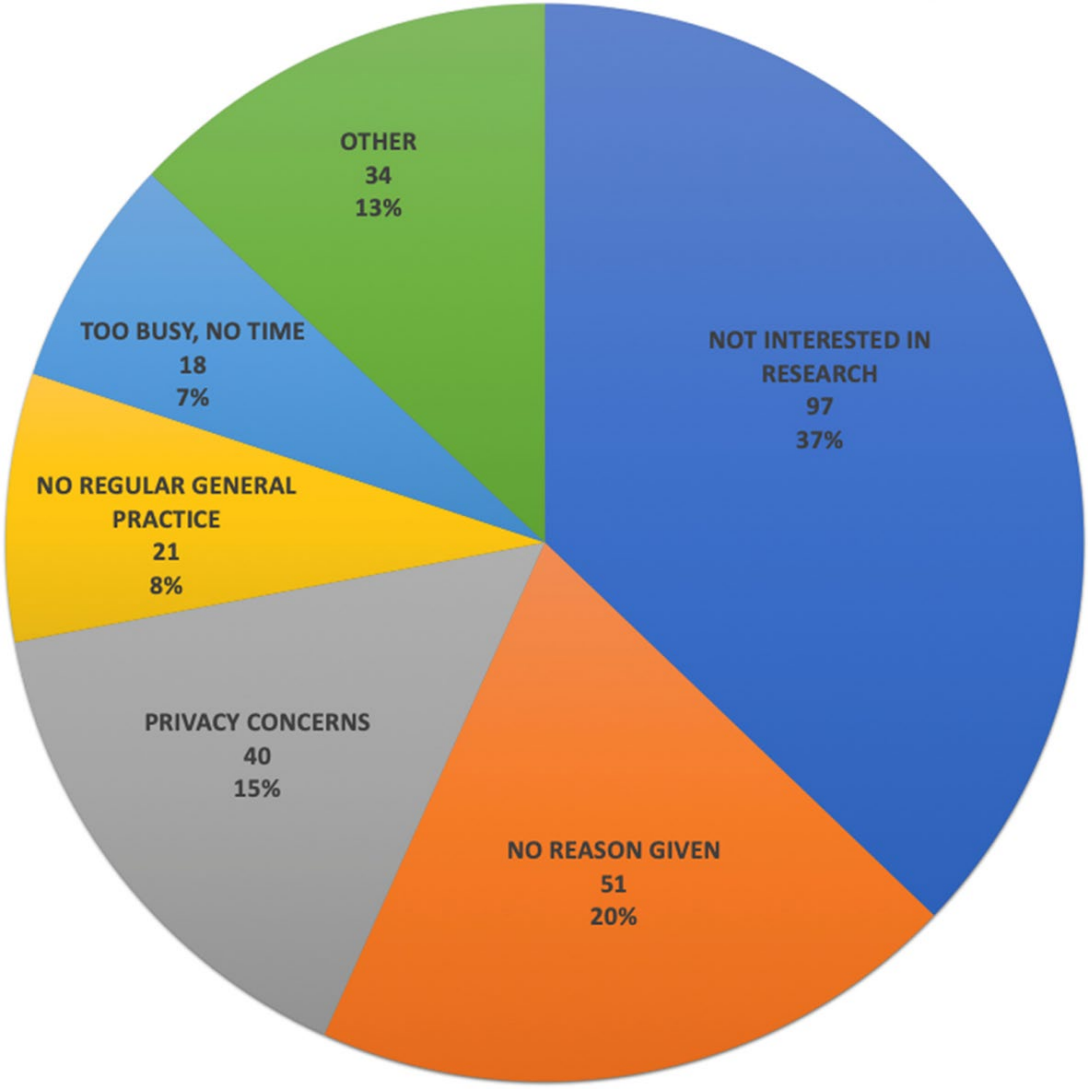
Number and proportions of participants in each category of non-consent to primary health care data extraction and linkage for research (total *n* = 261). ‘Other’ category consists of: ‘Does not believe they had COVID-19’ *n* = 9; ‘Bad experience with COVID-19’ *n* = 5;’Treated badly by Queensland Health’ *n* = 4; ‘Live overseas’ *n* = 4; ‘Research study concept is not valid’ *n* = 4; ‘Ongoing health issues’ *n* = 3; ‘Feel over-researched’ *n* = 2; ‘Language barrier’ *n* = 1; ‘Job at risk if identified’ *n* = 1; ‘Patient recently deceased’ *n* = 1

The main reasons for non-consent are shown in Fig. 2. The commonest categories were ‘not interested in research’ (37%), ‘concerns about privacy’ (15%), ‘no regular GP’ (8%) and ‘too busy/no time’ (7%). ‘No reason’ was given for non-participation in 20%. For those who were categorised as ‘not interested in research’, this included those who verbally stated they were not interested in participating (almost all), or texted the response ‘No’ when the liaison team member sent a short message reminder asking if they were interested in taking part in the research study.

Most participants who declined due to privacy concerns did not give a specific reason, simply stating “I am not prepared to have my medical records accessed” or “I don’t want to give access to my health record.” For those who did provide a reason, some were “worried about being identified in the community” and others did not want their whole medical record released—“thank you for the offer, however I do not feel comfortable releasing my full medical record.” A small number were concerned about the ability of Queensland Health to securely store their records.

In the group that were too busy to participate, reasons given included: “I don’t have time to go back and forth with phone calls,” “I’m super-busy at work,” or “I’ve too much on my mind and I don’t want anything extra.” One patient stated they would participate if paid.

In the ‘other’ category, 9 participants did not believe they had the virus despite testing positive for COVID-19. Some noted they had a bad experience with COVID-19 and did not wish to participate because the memory was too distressing; “I want to put COVID behind me, I have had several family members die overseas and it still rattles me”, “COVID has ruined my life” or “I just want it all to be over and not think about it”.

A small proportion of participants felt badly treated by Queensland Health as part of the COVID-19 pandemic response, such as “my case was handled so badly that I am considering legal action.” Other reasons for not consenting to data release were that Queensland Health in the past did not provide sufficient information to them and therefore “now the shoe was on the other foot.” Only a small number felt the study was not valid pointing out that there were “higher quality studies in countries with more COVID cases” or “likely small sample size and could not produce useful results” or just a “mass study on flu.”

## Discussion

This study found that two-thirds of participants who reached a consent decision gave consent for health information linkage for research. A slightly higher proportion also consented to recontact to discuss clinical trial participation. In those who declined, the most common reasons given for not consenting to link primary care data for research were a lack of interest in research, concerns around privacy, and not having a regular GP. People most likely to consent were those who were older and less socioeconomically disadvantaged.

This study differs from the majority of previous reports as it investigates why participants decline to take part in data linkage research, rather than tending to explore reasons they might agree [17]. In a similar study to ours, the activities taken up by people in retirement were explored using a cross-sectional survey [22]. Those who declined to answer the survey were invited to give their reasons for non-consent. Consistent with our study, 28% of the decline group reported themselves to be ‘not interested in research’. The investigators suggested that study misinterpretation may have contributed to non-consent as 40% of the decline group appeared to have misunderstood the purpose of the study. Poor understanding of the concept of data linkage and its value for health research by the public has been demonstrated to a be a common issue [22, 30–33]. One study found that once participants were educated and understood the value of data linkage, they transitioned to a willingness to share data [30]. This suggests that a clear explanation as to the meaning and value of health care data linkage to prospective participants may improve consent rates.

Concern about privacy is a common theme cited in many reports as to why participants do not wish to share their health data [17, 34, 35]. Specific reasons include inadequate data security and subsequent exploitation by companies to discriminate and make profit [34]. However, evidence suggests that concerns around privacy can be mitigated by being transparent about privacy protection measures in place and providing clear assurances of confidentiality [35]. Good governance, accountability, trust in the organisation managing their data, use of ethics, and legitimate use of data for the public benefit, have also been shown to be important reassurances [35].

Only a small subset of participants in our study opted out of participation due to not having a regular GP, resulting in the unavailability of primary healthcare data for export. This is most likely due to the vast majority of the public in Australia have attended a GP at some point in their lifetime and over 80% of the Australian population visit their GP at least once per year [36].

Previous studies investigating patient attitudes to linkage studies have not mentioned time constraints as a factor that discourages participation. This is because, apart from providing initial consent, health data sharing usually requires little involvement on the patient’s behalf. Our study may have invoked such a response because the consent process required two telephone calls and the patient having to access their email and return a consent form. It is also possible that patient misunderstanding and overestimation of the personal time required to participate in data linkage research may have contributed [22].

A significant proportion of participants did not give a reason as to why they did not wish to participate. The proportion not giving a reason may have been lower if the GP liaison team had been able to directly question these participants, but this was beyond the scope of the study.

Participants’ ages are reported to affect attitudes to consent. The general findings are that older respondents are more willing to share or link data than younger participants [17]. However, there is some variation, mostly by country in which younger participants have higher consent rates, making direct comparison with Australia difficult [26–29]. Gender differences in consent rates have not been consistently found, but when identified to be a predictor, males were more likely to consent [17, 27]. Participants with the highest socioeconomic status in our study were more than two-fold more likely to consent compared to the lowest category. Two other studies present divergent findings regarding the relationship between socioeconomic status and consent. While one study aligns with our results, demonstrating similar findings, the other study indicates no significant association between socioeconomic status and consent [28, 29]. Our study did not find that living in a more remote area affected consent levels, which differs with other reports which noted that increasing remoteness was associated with lower consent levels [28].

Three percent of participants declined to participate because they did not believe they had COVID-19 despite testing positive for COVID-19 and appearing as a COVID-19 case in the notifiable conditions surveillance system. Although there is much published in the literature about COVID-19 disbelief and vaccine hesitancy, there is little published on the proportion of participants who test positive for the virus yet do not believe the result. Importantly, only a small proportion of participants believed the study was invalid suggesting that the public understand the value of healthcare data linkage.

If some of these barriers to consent were addressed, the proportion agreeing to having their data linked could be increased. Many studies have found that participants are more likely to consent to data sharing if they are given choice, transparency, feedback, and ongoing control over what happens to their data [13, 30, 35, 37]. One method of meeting all of these requirements is through the use of dynamic consent [19]. Dynamic consent is a novel, entirely self-intuitive, web-based accessible consent platform which can be electronically sent to, and used by, participants on their desk-top or hand-held devices. Dynamic consent promotes an active ‘two-way’ patient-researcher interaction for greater patient participation in their own health and medical research. Patient participation is achieved by providing updateable consent choices, feedback as to when their data was used and published, along with regular posts on new research opportunities. Dynamic consent permits access to, and communication with, a much greater proportion of the population—including those living in rural, regional, and remote areas. Although a relatively new concept, several studies are implementing dynamic consent particularly in the area of genetics research [38, 39].

### Limitations

This study only included those participants who had been diagnosed with COVID-19 and therefore applying the results of this study (external validity) to other data linkage studies needs to be considered. The categorisation of responses given by participants were, to a certain degree, open to subjective interpretation by the GP liaison team, however this was mitigated by asking the team to record all verbal responses by participants.

## Conclusion

This study showed that the majority of participants who reached a consent decision are willing to consent to health care data linkage for research and permission for recontact to discuss clinical trial participation. Older participants and the least socio-economically deprived are more likely to participate. The study also sheds light on the reasons participants decline. Lack of patient interest in research, time required to participate and privacy concerns, were the most common reasons cited for non-participation. For future health care data linkage studies and strategies to increase clinical trial recruitment, addressing these issues may prove helpful.

## Data Availability

Datasets used and analysed during this study are available from the corresponding author upon reasonable request.

## Abbreviations

ACV19: ATHENA COVID-19
ATHENA: Australians Together Health Initiative
COVID-19: Coronavirus disease 2019
GP: General practice
NOCS: Notifiable Conditions System
PICF: Patient Information Consent Form
PHN: Primary Health Network
ARIA +: Accessibility and Remoteness Index of Australia
SEIFA IRSD: Socio-Economic Indexes for Areas, Index of Relative Socio-Economic Disadvantage
